# Prediction of the Potential Geographical Distribution of the Woolly Whitefly *Aleurothrixus floccosus* (Maskell) on Sweet Orange

**DOI:** 10.1002/pei3.70169

**Published:** 2026-06-01

**Authors:** Gelana Keno Beyene, Emana Getu, Tarekegn Fite, Ashenafi Kassaye, Muluken Goftishu, Megersa Kebede

**Affiliations:** ^1^ School of Plant Sciences, College of Agriculture and Environmental Sciences Haramaya University Haramaya Oromia Ethiopia; ^2^ Department of Plant Science, College of Agriculture Oda Bultum University Chiro Ethiopia; ^3^ Department of Zoological Sciences Addis Ababa University Addis Ababa Ethiopia; ^4^ Bako Agricultural Research Center, Oromia Agricultural Research Institute Bako Ethiopia

**Keywords:** climatic variables, invasive pest, MaxEnt, pest forecast

## Abstract

*Aleurothrixus floccosus* is an exotic pest that poses a serious threat to sweet orange, both through direct and indirect damage. Climate change is one of the most important factors that facilitate the distribution and occurrence of species. We focused on the Ethiopian context because there was no localized data for this invasive pest. Thus, the Maxent (Maximum Entropy) model was used to predict the potential distribution of *A. floccosus* under the current and future climatic situations in Ethiopia. The occurrence data were obtained from field surveys, and bioclimatic variables (bio1–bio19) were loaded from the Global Climate Database. Among bioclimatic variables (bio1–bio19), precipitation of the driest month (bio14), precipitation of the warmest quarter (bio18), precipitation of the wettest month (bio13), precipitation seasonality (bio15), mean diurnal range (bio2), isothermality (bio3), and temperature annual range (bio7) were key predictors of *A. floccosus* distribution, with contributions of 28.2%, 22.9%, 21.8%, 17.7%, 4.7%, 3.9%, and 0.8%, respectively. Very highly suitable areas for *A. floccosus* are mainly intense in the Central Ethiopia Regional State, Gambela Region, Dire Dawa city, Harari Region, and large parts of the Oromia Region. Models from RCP2.6, RCP6.0, and RCP8.5 (2050 and 2070) predict that very highly suitable areas for *A. floccosus* increase from 0.32% to 3.52% under future climate conditions. Unsuitable, poorly, moderately, highly, and very highly suitable areas for *A. floccosus* covered 40.40%, 22.80%, 20.90%, 10.60%, and 5.30%, respectively, of the total Ethiopian landmass. However, further studies are needed to assess other possible factors affecting *A. floccosus* distribution.

## 1 Introduction

Woolly whitefly, *Aleurothrixus floccosus* (Maskell), causes a serious threat to sweet orange producers, both through direct and indirect crop damage (Da Silva et al. 2024). *A. floccosus* direct injury happens when they feed and insert stylet into plant phloem and continuously suck nutrients, causing twig infestation (80%–100%), leaf infestation (60%–99%), and fruit infestation (35%–37%) (Nega et al. 2014). Indirect damage by the pest occurs through droplets and honeydew production by the insect that accumulates dust and creates favorable conditions for the growth of black sooty molds, which can completely cover the leaf and fruit sides and interfere with the photosynthetic activity of the plant, such as respiration and transpiration, and then cause qualitative deterioration in fruit resulting in great economic impact on sweet orange (Soto et al. 2002; Belay et al. 2011). The woolly whitefly produces abundant amounts of sticky honeydew at the lower part of leaves of citrus trees (Grout et al. 2012). The infestation by *A. floccosus* nymph was rated more than 80% during the warm season and reduced to 20% in the cool season in Northwestern Algeria (Mahmoudi et al. 2018). At the nymphal and adult stages, they suck the phloem sap (fluid) and cause heavy infestations resulting in leaves falling (Giliomee and Millar 2009).

The growth and development of life organisms are closely associated with climatic variables, which may affect the form of biodiversity (Zhang et al. 2018). Climate change is one of the greatest important factors that facilitate the dispersal, occurrence, and growth of the *A. floccosus* through increasing temperatures and changing rainfall patterns that optimize its development while usually disordering its natural enemies (Laštuvka 2009; Tomson et al. 2010; Soto et al. 2002). Global warming is characterized as a major problem in the twenty‐first century (Guo et al. 2019). Climate change, such as extreme climatic variables, intensely influences species distribution and habitat appropriateness and could boost invasive procedures from their origin spread to other extra possible geographical areas (Diez et al. 2012; Welter et al. 2003). Because of the alterations in the agroclimatic conditions of different provinces, arthropods exhibit fluctuating manners in terms of their incidence and the amount they cause damage to crops (Elango and Nelson 2021).

*A. floccosus* initially emerged as a citrus threat around 1909 in Tampa, which is located in South America (Nega et al. 2014). This invasive pest's first appearance in Africa was in Kenya in 1990, after which it quickly disseminated across citrus‐growing areas of the landmass, including Ethiopia (Nega et al. 2014). In Ethiopia, the occurrence of *A. floccosus* in the rift valley areas was reported for the first time by Emana (2003). This pest causes infestations to all citrus species in Adama town, Debrezeit town, Zewayi, Arsi‐Negele, Sheshemene, Adami Tulu, Meki, and Ambo and their surroundings of citrus growing areas (Getu 2007; Nega et al. 2014).

The biology of the pest was reported by Giliomee and Millar (2009), who said that at 22.5°C, to reach adulthood after hatching from the egg, it took 27.4 days and passed over four stages nymph. Adults live for periods of 36 days, and they can lay an average of 53 eggs during their lifespan. *A. floccosus* adults are characterized by wings covered by a white waxy substance, and their color is yellowish white and found hidden on the underside of completely extended leaves, and lay eggs on the bottom of the leaves in a circle, with each round egg enclosed by a light dusting of waxy substances (Getu 2007). The immature stages are flattened, oval‐shaped, and resemble scales (Kerns et al. 1996). The adult male size is somewhat smaller than the female, reaching about 1.5 mm (Tello Mercado et al. 2014).

Pest prediction, detection, and early warning or giving attention are important mechanisms of the integrated pest management approach (Dara 2019). Many species distribution models, for example MaxEnt, BIOCLIM, CART, ENFA, GLM, and GAM, have emerged and are commonly used in the fields of biogeography, ecology, and conservation biology (Gaston 1996). Among these, the MaxEnt (maximum entropy model) shows good stability, and the distribution area of the species can be correctly forecasted though the distribution data and environmental variables of the distribution area are inadequate (Guisan and Thuiller 2005; Booth et al. 2014; Yang et al. 2025). It is also a widely used correlative model in forecasting or mapping the potential distribution of alien species (Da Silva et al. 2017; Kumar et al. 2014). Maxent has advantages such as supporting small sample sizes, performing jackknife tests, using only presence records, high flexibility, accurate predictions, and easy result interpretation (Yuan et al. 2015). Rapidly detecting climatic suitability (risk of establishment) of invasive insect species before they can cause significant economic damage can help to detect and slow their spread, prevent their establishment, and manage existing populations more sustainably and economically (Tonnang et al. 2017). Having clear information on the habitat suitability of a target strange species can support interventions and the development of integrated pest management programs (Barkessa et al. 2025).

We focused on the Ethiopian context rather than Africa or the world because there was no localized data for this invasive pest. Insect prediction is usually more reliable in smaller areas because it allows for more comprehensive data collection and the use of more intensive models. No studies have yet been performed to forecast the ecological suitability of invasive *A. floccosus* under climate change conditions in Ethiopia. Thus, this study was done to forecast the possible distribution of *A. floccosus* in Ethiopia under current and future climate changes to detect the main bioclimatic variables influencing the distribution of the pest through an ecological niche model‐based approach and to share information with concerned bodies in the country to come up with a solution for this pest invasion.

## 2 Materials and Methods

### 2.1 Woolly Whitefly, *Aleurothrixus floccosus* Occurrence or Presence Data

The occurrence or presence data of the *A. floccosus* (Maskell) were taken from field surveys in open‐field sweet orange fruit farms across major citrus‐producing areas of Ethiopia. Geographic coordinates of latitude and longitude of current pest occurrence were recorded (Table 1).

**TABLE 1:** Details of *A. floccosus* occurrence data.

Region	Zone	District	Sample site name	Latitude (N)	Longitude (E)
Oromia	East Shewa	Adama town	Adama	8°54′	39.27°
		Bishoftu town	Bishoftu	8°75′	38.983°
		Wonji town	Wonji	8°31′	39°12′
		Melkasa Research Center	Malkassa	8°24′	39°21′E
		Dugda	Maki	8°9′	38°49′
		Batu town	Batu	7°56′	38°43′
		Mojo town	Mojo	8°39′	39°5′
		Adama	Sodare	8°24′	39°23′
		Koka town	Koka	8°26′ 3.92	39° 1′ 50.8
		Adama	Kuriftu resort	8.7347°	39.0085°
		Jido kombolcha	Adami tulu	7.8648°	38.7080°
		Jido kombolcha	Bulebula	7.7195°	38.6501°
		Boset	Nura Hera	8°36′2.88	39°43′6.96
		Boset	Dire degaga	8.49	39.42
	West Shewa	Bako tibe	Tibe	9°4′12.00	37°8′60.00
		Bako tibe	Bako	9°08′	37°03′
		Ambo	Ambo	8°59′	37°51′
	West Hararghe	Daro labu	Mechara	8.6018034	40.3343827
		Daro labu	Agamti	8°26′2″	40°26′3″
		Daro labu	Haroresa Kile	8°26′16″	40°22′52″
		Daro labu	Madhisa	8°27′12″	40°22′34″
		Daro labu	Haroresa Huluko	8°27′27″	40°22′24″
		Daro labu	Satawa	8°28′42″	40°21′47″
		Daro labu	Haro Abdi	8°29′4″	40°20′49″
		Doba	Biftu Oromia	9°13′15″	40°57′20″
		Doba	Tokuma	9°11′40″	40°58′26″
		Tulo	Ragasis	9°10′0″	41°6′30″
		Tulo	Gemachis	9°8′21″	41°6′45″
		Tulo	Ganda daga	9°11′38″	41°6′23″
	East Hararghe	Malka ballo	Daga balo	9°0′5″	41°23′38″
		Malka ballo	Dire kufa	9°4′42″	41°21′59″
		Malka ballo	Mulisa	9°1′4″	41°23′33″
		Malka ballo	Biftu naga	9°0′42″	41°23′41″
		Deder	Hufe	9°3′44″	41°21′26″
		Deder	Jirme	9°3′19″	41°21′15″
		Deder	Ifa bas	9°4′27″	41°21′36″
		Babile	Sheck abdi	9°21.07	42°35.19
		Babile	Fetiya farm	9°13′39″	42°15′44″
	West Wollega	Nejo	Nejo town	9°30′	35°30′
		Mana sibu	Mendi twon	9°36′	35°36′
		Begi	Begi town	9°20′	34°29′
		Kondala	Kondala	9°14′60	34°44′59.9″
	East Arsi	Diksis	Dhera	8°20′	39°31′
		Jeju	Jeju	8°19′60.00″	39°09′60.00″
		Merti	Merti	8.51	39.86
		Tiyo	Tibila	8.5	39.5667
	West Arsi	Arsi negele	Negele	7.3599°	38.6709°
		Shashemane	Shashemane	7.2010°	38.6065°
	Special zone	Dukam	Dukam	8°48′31	38°54′52
	Jima	Gera	Jare	8°8′56.04	35°32′12.8″
		Agaro	Agaro	7°51′0″	36°39′0″
		Jima	Jima university	7°41′5″	36°49′53″
	Bale	Berbere	Ledi chekata	7.91145	41.31943
		Berbere	Haro nano one	8°28′0″	38°37′0″
		Berbere	Haro nano two	6.76°	40.30
		Berbere	Haro dumal	7.5460°	40.6346
		Berbere	Sirima	6.5°	39.9°
		Bale robe	Awuraris	7.719	40.005056
	South west Shewa	Goro	Goro, waliso	8.4006°	37.8685
Sidama	Aleta wendo town	Aleta wendo	Aleta wendo	6°35′59.99	38°24′59.99
South western part of Ethiopia	Keffa	Bonga	Bonga	7.2672	36.2468
	Benchi Maji	Sheko	Bench sheko	6.0620	35.5658°
	Keffa	Ginbo	Ginbo	7°19′60	36°09′60
Dire Dawa	Dire Dawa	Dire Dawa	Tony farm	9°36′53″	41°50′25″
		Dire Dawa	Melka jabdu	9°37′2″	41°47′5″
		Dire Dawa	Asegedech farm	9°36′3″	41°51′26″
		Dire Dawa	Dire Dawa 03	9°35′7″	41°51′38″
		Dire Dawa	Jallo balina	9°53.32′	41°87.87′
		Dire Dawa	Dire Dawa 02	9°61.20′	41°84.16
Ahmara	North shewa	Efratana Gidim	Shewarobit	10°00′	39°54′
	West gojjam	Ataye	Ataye	10.35	39.96
		Finote selam	Finote selam	10°42′	37°16′
	Agew Hawi	Guangua	Chagni	10°57′	36°30′
	South Wollo	Kalu	Harbu	10°55′12″	39°47′13″
		Dawa Chaffa	Kemise	10°43′	39°52′
	North Wollo	Waldiya	Waldiya	11°49′33	39°35′33
Tigray	Central	Tanqua Millash	Agibe	13.54955	39.05152
		Kola Tembien	Adiha	13.7456°	39.1336°
		Abergelle	Sheka Tekli	13°14′06″	38°58′50
Harari	Abadir	Abadir	Abadir	9.3010°	42.1424°
	Erer Kile	Erer Kile	Erer Kile	9°14′33″	42°14′48″
	Harewae	Harewae	Harewae	9.25.63′	42°05′98″
Afar	Administrative	Awash Fentale	Sabure	11°45′21.38	40°57′31.28
		Fantale	Awara Melka	9.14326	39.96657
South Ethiopia Regional State	Gamo	Arbaminchi	Arbaminchi	6°2′	37°33′
Somali	Shinile	Erer Gota	Kantaras	9°32′11″	41°23′57″
		Erer Gota	Gode	9°34′8″	41°23′25″
		Erer Gota	Bila	9°32′22″	41°26′5″
		Erer Gota	Dimtu	9°36′36″	41°38′45″
		Erer Gota	Hurso	9°33′54″	41°31′2″

For confirmation of the pest, the collection of infested sweet orange plant parts was sampled. About 90 total occurrence data in the country were collected. The occurrence data were used when the presence of *A. floccosus* was confirmed by visual or official reports. The geographic coordinates for each occurrence information were compiled via the global positioning system (GPS), and duplicate neighboring occurrence records were removed at a resolution of < 10 m based on the Maxent model (Jin et al. 2025). The data were inspected visually to check and remove duplicate records and were also cleaned via Maxent software. Lastly, a total of 80 occurrence records were used to create the distribution model of *A. floccosus* in Ethiopia. In total, 80 occurrence screened records were used because the sweet orange production area in Ethiopia is very low and limited to some places.

### 2.2 Environmental Variable Data

The bioclimatic variables (bio1–bio19) at 30 arc‐second approximately 1 km resolution were evaluated from the Global Climate Database (https://www.worldclim.org) to build up a species distribution model of suitable areas for *A. floccosus* under the current and future climatic conditions in Ethiopia. Bio 1 = Annual Mean Temperature, bio 2 = Mean Diurnal Range, bio 3 = Isothermality, bio 4 = Temperature Seasonality, bio 5 = Max Temperature of Warmest Month, bio 6 = Min Temperature of Coldest Month, bio 7 = Temperature Annual Range, bio 8 = The Mean Temperature of Wettest Quarter, bio 9 = The Mean Temperature of Driest Quarter, bio 10 = Mean Temperature of Warmest Quarter, bio 11 = Mean Temperature of Coldest Quarter, bio 12 = The Annual Precipitation, bio 13 = The Precipitation of Wettest Month, bio 14 = Precipitation of Driest Month, bio 15 = Precipitation Seasonality, bio 16 = Precipitation of Wettest Quarter, bio 17 = Precipitation of Driest Quarter, bio 18 = Precipitation of Warmest Quarter and bio 19 = Precipitation of Coldest Quarter were used. The occurrence datasets of *A. floccosus* were saved in Microsoft Excel. Pearson correlation analysis was made on the extracted variables via SPSS software to evaluate the size of spatial autocorrelation among the variables to cut out redundant information and progress prediction accuracy.

### 2.3 Modeling Approach

The Maxent model was used to forecast the potential distribution of *A. floccosus* pest occurrence. To avoid collinearity between modeling variables affecting the prediction results, the variables were screened as per the author of Wang et al. (2020) procedures. 1st, the initial model was formed by importing both the occurrence points of *A. floccosus* and 19 bioclimatic variables into the Maxent model. The percent contribution and permutation contribution of each separate variable to the primary simulation results were determined via a jackknife test to select the core environmental features for the prediction. To avoid variable spatial autocorrelation, a Pearson correlation analysis was performed on the filtered variables via SPSS version 22. Based on the percent involvement of each bioclimatic variable with correlation coefficients of > 0.8 (highly correlated), they were filtered. After this method of selection, seven bioclimatic variables were chosen as potential predictors of *A. floccosus* habitat suitability, namely, the mean diurnal range (bio 2), isothermality (bio 3), precipitation of the warmest quarter (bio 18), annual temperature range (bio 7), precipitation of the wettest month (bio 13), precipitation of the driest month (bio 14), and precipitation seasonality (bio 15).

### 2.4 Distribution Modeling

The seven screened bioclimatic variables were used to determine the relationships between the possible distribution of *A. floccosus* and current climate circumstances. The occurrence areas of *A. floccosus* and the seven bioclimatic variables were fed into the Maxent to simulate the dispersal of *A. floccosus* in Ethiopia. Model replicates were run ten times to improve model performance, and the finest model with the maximum AUC (area under the curve) value was preferred to assess the probability distribution of the pest (Xu et al. 2019). The results from the Maxent model were imported into ArcGIS and changed into a raster format file via “Conversion Tools”, which was used to categorize and visualize the distribution areas of the pest, and the “Extraction” function was used to produce a graphical illustration of the pest distribution across Ethiopia.

### 2.5 Model Justification and Visualization

The receiver operating characteristic (ROC) curve and the area under the ROC curve (AUC) are very useful and usually used tools for evaluating the discriminative power of a species distribution model (Wang et al. 2020). The area under the ROC curve (AUC), which varies from 0.5 to 1, was taken as a measure of model accuracy, with a value close to 1 telling high simulation accuracy (Araújo et al. 2005). For a value of 0.5 ≤ AUC < 0.5, the evaluation criteria recommend prediction failure; 0.6 ≤ AUC < 0.7, poor prediction results; 0.7 ≤ AUC < 0.8, fair prediction results; 0.8 ≤ AUC < 0.9, good prediction results; and 0.9 ≤ AUC < 1, excellent prediction results (Tenguri and Shah 2017).

## 3 Results

### 3.1 Role of Bioclimatic Factors

The choice of climatic variables is the foremost reason for the accuracy of the results. The percent involvement of each bioclimatic variable to the distribution simulation model was numeric via the knife‐cutting technique (Table 2), which shows that Precipitation of Driest Month (bio 14) completes the greatest contribution to the predicted distribution of *A. floccosus* (28.2%), followed by Precipitation of Warmest Quarter (bio 18), Precipitation of Wettest Month (bio 13), and Precipitation Seasonality (bio 15), with contributions of 22.9%, 21.8%, and 17.7%, respectively. The factors diurnal range (bio 2), isothermality (BIO2/BIO7) (× 100) (bio 3), and annual temperature range (BIO5–BIO6) (bio 7) made minor effects, with values of 4.7%, 3.9%, and 0.8%, respectively. Variables that did not illustrate any contribution were removed.

**TABLE 2:** Comparative percentage of bioclimatic variables to forecast the distributions of *A. floccosus* .

Environmental variables	Percent contribution (%)	Permutation importance (%)
bio14	28.2	20.7
bio18	22.9	8.5
bio13	21.8	26.4
bio15	17.7	40.1
bio2	4.7	3.5
bio3	3.9	0.1
bio7	0.8	0.7

### 3.2 Relationships Among the Bioclimatic Variables

There were negative correlations between isothermality (BIO2/BIO7) (× 100) (Bio3) and temperature annual range (BIO5–BIO6) (bio7) with a value of −0.33, temperature annual range (BIO5–BIO6) (bio7) and precipitation of the driest month (bio14) with a value of −0.06, precipitation of the driest month (bio14) and precipitation seasonality (bio15) with a value of −0.68, and precipitation of the warmest quarter (bio18) and precipitation seasonality (bio15) with a value of −0.41. As the annual temperature range (BIO5–BIO6) decreases, isothermality (BIO2/BIO7) (× 100) increases. As the temperature annual range (BIO5–BIO6) increases, the precipitation of the dehydrated month decreases. A positive correlation was found between the other environmental variables except for the above negatively correlated variables (Table 3).

**TABLE 3:** Relationship of the bioclimatic variables helped to construct the *A. floccosus* distribution model.

Variables	Bio2	Bio3	Bio7	Bio13	Bio14	Bio15	Bio18
Bio2	1.00	0.48	0.67	0.60	0.19	0.05	0.36
Bio3	0.48	1.00	−0.33	0.22	0.32	0.03	0.43
Bio7	0.67	−0.33	1.00	0.45	−0.06	0.04	0.03
Bio13	0.60	0.22	0.45	1.00	0.21	0.12	0.23
Bio14	0.19	0.32	−0.06	0.21	1.00	−0.68	0.48
Bio15	0.05	0.03	0.04	0.12	−0.68	1.00	−0.41
Bio18	0.36	0.43	0.03	0.23	0.48	−0.41	1.00

### 3.3 Modeling Performance

**FIGURE 1:**
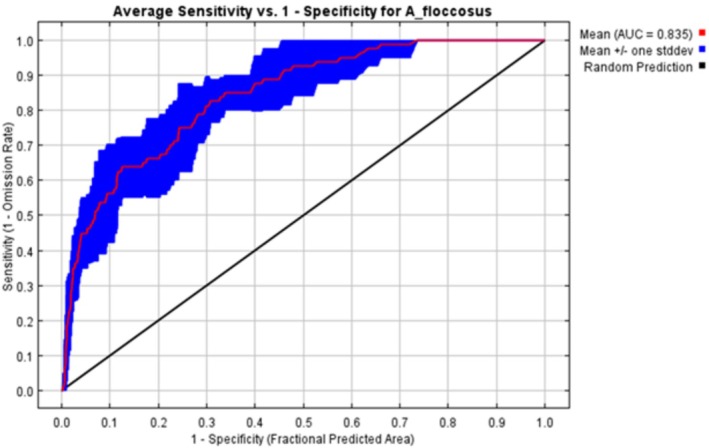
The receiver operating characteristic curve modeling.

The analysis result of ROC showed that the value of the training dataset was 0.835 (Figure 1). Compared with the corresponding random prediction models (AUC = 0.5), the AUC value obtained for the model predicting the 10 replicates was 0.835, which falls within the range of 0.8–0.9, indicating that the model performed well in terms of prediction.

### 3.4 Jackknife Examination of Training Data

**FIGURE 2:**
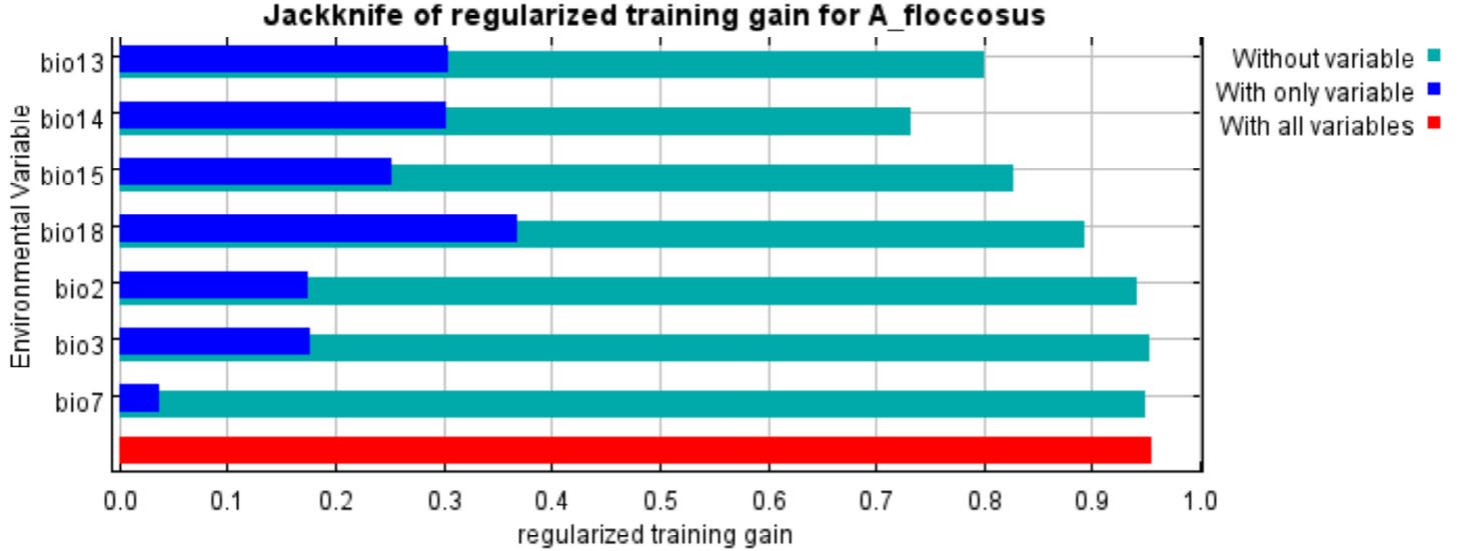
The comparative analysis of bioclimatic variables based on the Jackknife of regularized train gain of *A. floccosus*.

The jackknife results revealed that the variable with the greatest contribution was Precipitation of Warmest Quarter (bio 18), followed by Precipitation of Wettest Month (bio 13), Precipitation of Driest Month (bio 14), Precipitation Seasonality (bio 15), Isothermality (BIO2/BIO7) (× 100) (bio 3), and Mean Diurnal Range (bio2), which have the greatest impact on the model, followed by the temperature annual range (BIO5–BIO6) (bio 7), which has the least impact on the model (Figure 2). In the present study, Precipitation of Warmest Quarter (bio18) was the most important variable that contributed to predicting the potential distribution of *A. floccosus* in Ethiopia. The precipitation of the warmest quarter was compared with other climate variables to well understand the potential influence of future climate change. The environmental variable with the greatest gain when used in isolation was bio 18, which gives the idea of the greatest useful information by itself, whereas bio 7 has the least amount of information by itself (Figure 2).

### 3.5 Relationships Between Bioclimatic Variables and the Occurrence of *A. floccosus*

**FIGURE 3:**
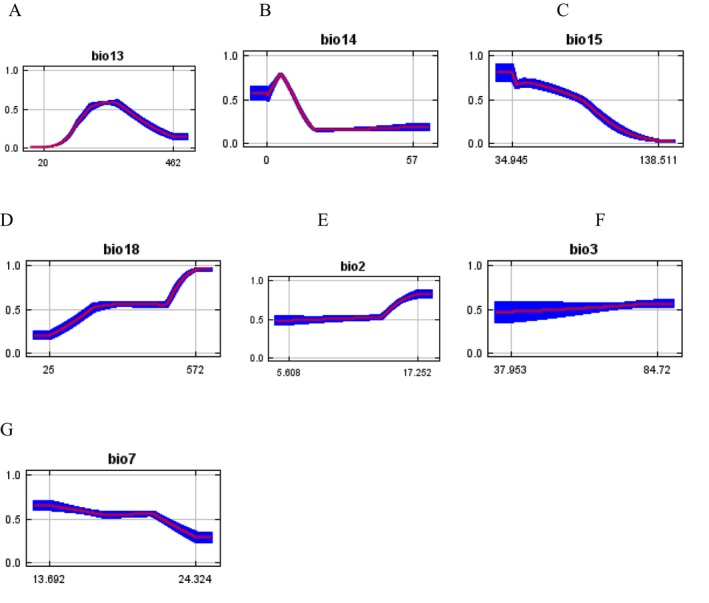
Climate changes influencing the distribution of *A. floccosus*.

The *A. floccosus* distribution increased with increasing values of each bioclimatic variable within a certain range and reduced after a certain highest (peak). On the basis of this analysis, the variable that contributed the most to high suitability was a precipitation of 25–572 mm (Figure 3D) for the Warmest Quarter precipitation (bio18). Other climate variables also displayed a relationship with a high probability of presence, namely, 20–462 mm (Figure 3A) for precipitation of wettest month (bio13), 0–57 mm (Figure 3B) for precipitation of the driest month (bio14), between 34.95 and 138.51 mm (Figure 3C) for precipitation seasonality (bio15), 5.61°C–17.25°C (Figure 3E) for the mean diurnal range (mean monthly) (bio2), between 37.95 and 84.72 (Figure 3F) for isothermality (BIO2/BIO7) (× 100) (bio3), and between 13.69°C and 24.32°C (Figure 3G) for the temperature annual range (BIO5–BIO6) (bio7). The results suggested that the maximum habitat suitability of *A. floccosus* occurred when the precipitation of warmest quarter was 572 mm (Figure 3D), the precipitation seasonality (coefficient of variation) was 34.945 mm (Figure 3C), the mean diurnal range (mean of monthly (max temp − min temp)) was 17.252°C (Figure 3E), the isothermality (BIO2/BIO7) (× 100) was between 37.953 and 84.72 (Figure 3F), the annual temperature range (BIO5–BIO6) was 13.692°C (Figure 3G), the precipitation of wettest months was between 20 and 462 mm (Figure 3A), and the precipitation of driest months was approximately 10 mm (Figure 3B).

### 3.6 Current Potential Distribution of *A. floccosus* in Ethiopia

**FIGURE 4:**
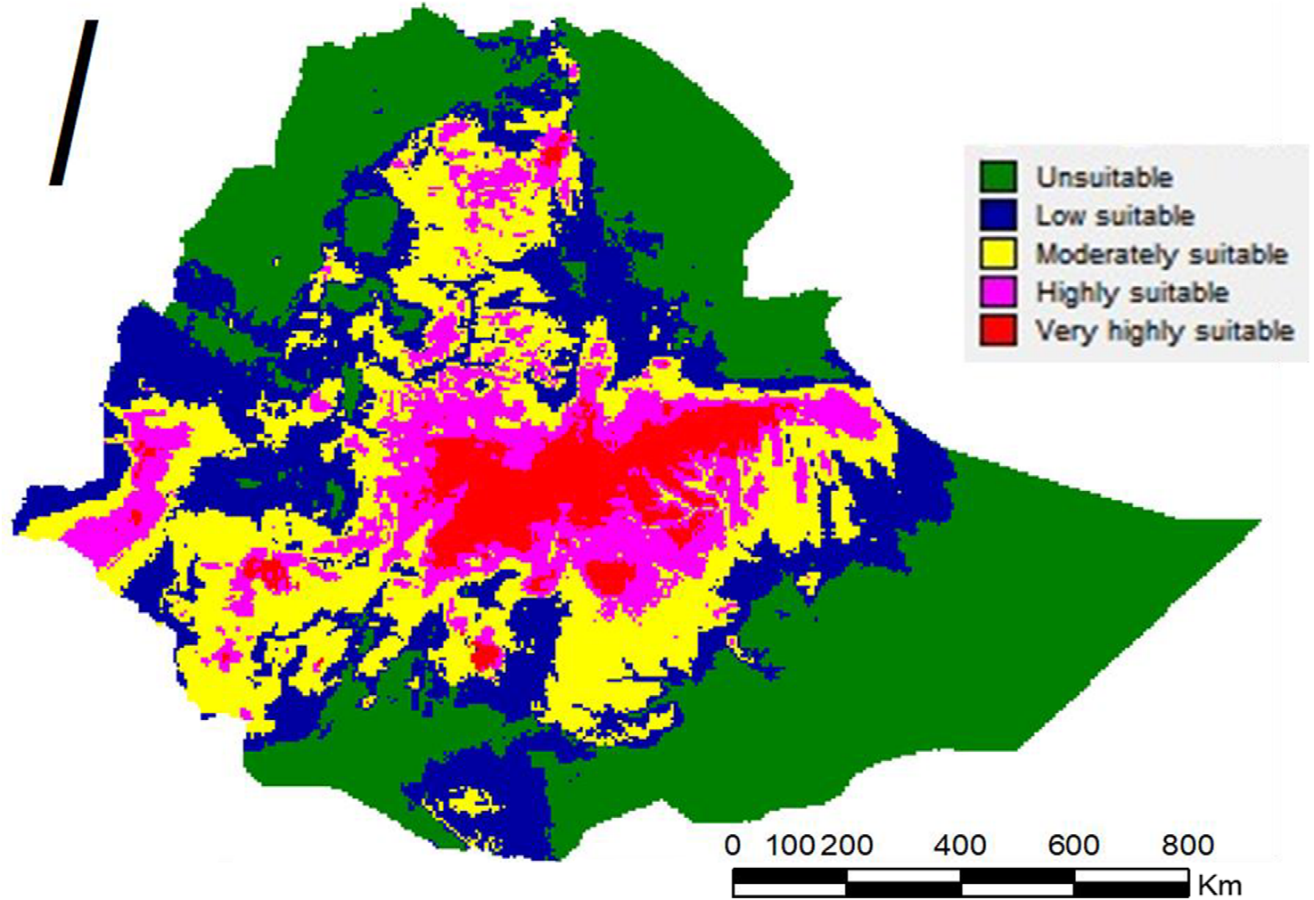
Current potential distribution of *A. floccosus* in Ethiopia.

The current potential distribution of *A. floccosus* determined via the Maxent model was created via DIVA‐GIS (Figure 4; Table 4). This result illustrated that the range of suitable habitat for *A. floccosus* was very widespread and that unsuitable areas were located in large parts of the Afar region, a small portion of the Amhara region, a very minor areas of the Oromia, a small areas of the South Ethiopia's regional state, large areas of the Somali region, and large of Tigray region containing an area of 45.65 × 10^4^ km^2^. The low‐suitability area was located mainly in some of the Afar regions, a small portion of the Amhara, some parts of the Benishangul‐Gumuz, a small portion of the Oromia, some portions of the South Ethiopia, a small portion of the Somali region, and some portions of the Tigray region, spanning an area of 25.80 × 10^4^ km^2^. The moderately suitable areas were mostly focused in Addis Ababa city, some of the Amhara regions, Benishangul‐Gumuz regions, some of the Oromia regions, Sidama regions, some of the Somali regions, some of the South Ethiopia regional states, some of the Tigray regions, and South West Ethiopia peoples, covering an area of 23.60 × 10^4^ km^2^. The highly suitable areas were distributed in most parts of the Amhara region and most parts of the Oromia regional state, covering an area of 11.94 × 10^4^ km^2^. The very highly suitable areas for *A. floccosus* in Ethiopia were mainly focused in the Dire Dawa city, Central Ethiopia Regional State, the Gambela Region, the Harari region, and most parts of the Oromia region, spanning an area of 60.09 × 10^4^ km^2^ (Figure 4; Table 4). The unsuitable habitats (40.40%), poorly suitable (22.80%), temperately suitable (20.90%), highly suitable (10.60%), and very highly suitable (5.30%) of *A. floccosus* comprised Ethiopian total landmass (Table 4).

**TABLE 4:** Forecasted suitable areas (km^2^) of *A. floccosus* under present and upcoming scenarios.

Period	Predicted area (10^4^ km^2^)						Comparison with current distribution (%)				
	Scenario	Unsuitable	Low suitable	Moderately suitable	Highly suitable	Very highly suitable	Unsuitable	Low suitable	Moderately suitable	Highly suitable	Very highly suitable
Current	—	45.65	25.80	23.60	11.94	60.09	—	—	—	—	—
	RCP2.6	45.69	26.53	23.21	11.53	60.28	0.09	2.74	−1.66	−3.48	0.32
2050	RCP6.0	44.26	25.98	24.47	12.20	60.77	−3.04	0.70	3.70	2.23	1.13
	RCP8.5	46.71	26.92	22.47	10.68	62.20	2.31	4.32	−4.77	−10.53	3.52
	RCP2.6	45.13	26.04	24.02	11.70	60.98	−1.14	0.94	1.80	−1.96	1.48
2070	RCP6.0	46.05	26.22	23.61	11.08	60.30	0.87	1.62	0.06	−7.13	0.35
	RCP8.5	45.14	27.12	23.76	11.12	58.61	−1.13	5.10	0.71	−6.87	−2.46

Abbreviation: –, not applicable.

### 3.7 Potential Future Distributions of *A. floccosus* Under Different Climatic Conditions

**FIGURE 5:**
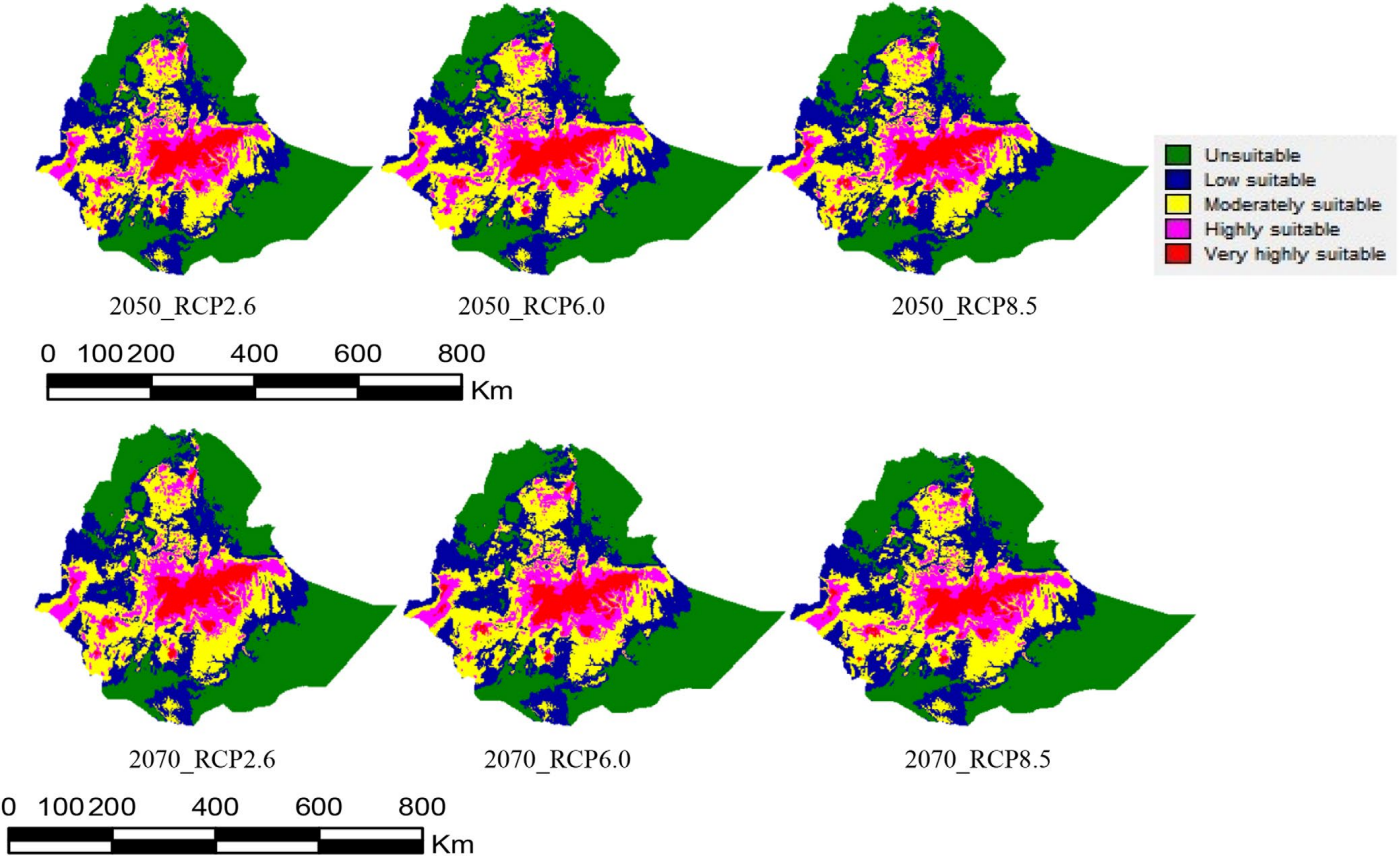
The future predicted distribution of *A. floccosus* under three common concentration pathways (RCPs 2.6, 6.0, 8.5) of climatic conditions in 2050 and 2070.

The results demonstrated that extensive differences existed between the current suitable habitat and that predicted for future periods, with moderately suitable areas and the continuous expansion of areas with low appropriateness and very high suitability. In the 2050s, the inappropriate area for *A. floccosus* under the RCP6.0 situation would reduce by 3.04% but rise by 0.09% and 2.31% under RCPs 2.6 and 8.5, respectively (Table 4 and Figure 5). The low‐relevance habitats would upsurge by 2.74%, 0.70%, and 4.32% under RCP2.6, RCP6.0, and RCP8.5, respectively. Very highly suitable areas would increase by 0.32%, 1.13%, and 3.52% under RCP2.6, RCP6.0, and RCP8.5, respectively, in the 2050s. For the 2070s, the area of inappropriate habitat for *A. floccosus* would decrease by 1.14% and 1.13% under RCPs 2.6 and 8.5, respectively, but increase by 0.87% under RCP 6.0. Moderately suitable places would increase by 1.80%, 0.06%, and 0.71% under RCP2.6, RCP6.0, and RCP8.5, respectively. Very highly suitable areas would increase by 1.48% and 0.35% under RCP2.6 and RCP6.0, respectively, in the 2070s (Table 4 and Figure 5).

## 4 Discussion

The detection of *A. floccosus* in Ethiopia has raised a serious concern about the potential distribution of this insect pest across citrus‐growing areas of the country. To address this issue, we predict for the first time to estimate the most suitable areas under current and future climate change conditions where the species may rapidly spread using the Maxent modeling approach to limit their expansions. The MaxEnt model has proven to be a critical tool for identifying suitable areas for surveillance and monitoring of invasive species by combining occurrence records with bioclimatic variables to predict potential distribution and spread, thereby supporting early detection and management strategies (Phillips 2006). The model reported in this study has good performance, with a value equal to 0.835 for the AUC. A model with a lower AUC value is less reliable, and a model with a large area under the ROC curve indicates that the model is better able to surely estimate the appearance and non‐appearance of the pest (Tenguri and Shah 2017).

*A. floccosus* develops rapidly in areas with conducive weather conditions, especially where humid conditions exist and where the temperature is more than 14°C, which permits constant oviposition throughout the period and highly damages the commodity (Rodas‐Martínez et al. 2023). Thus, from our result, it was confirmed in Ethiopia that most sweet orange‐growing areas where the presence of this pest was recorded were characterized by hot and humid weather conditions, which are most suitable for the rapid growth and development of whiteflies. Da Silva et al. (2024) reported that orange trees and other citrus trees prefer conditions of temperatures 23°C–32°C and high relative humidity (mostly 75%–80%). The optimum rainfall for citrus orchards is between 1000 and 1800 mm, demonstrating that the precipitation fluctuations influence the establishment of citrus cultures and insect pests in the same way; but, in places where there is a water deficiency, this problem can be reduced via irrigation (Da Silva et al. 2024).

As reported in these results, climatic factors such as isothermality (BIO2/BIO7) (× 100), mean diurnal range (mean of monthly (max temp − min temp)), temperature annual range (BIO5‐BIO6), precipitation of wettest month, precipitation of driest month, precipitation seasonality (coefficient of variation), and precipitation of warmest quarter were the seven major contributing variables for the potential distribution of *A. floccosus* in Ethiopia. Climatic change directly affects the reproduction, development, survival, and potential distribution of whitefly species (Bellotti et al. 2012; Gilioli et al. 2014). In agro‐ecosystems, the mean maximum temperature of 28.5°C and the mean minimum temperature of 10.8°C are favorable for the growth and lifecycles of *A. floccosus*, which produces seven generations in a year (France et al. 2011). Variations in rainfall level influence the *A. floccosus* population of developmental stages; results reduce the population when rainfall is high and increase the population of *A. floccosus* when favorable breeding conditions of the dry seasons (Salinas and Sumalde 1994; Meh and Deyemi 2011). As Soliman (2023) reported in the context of global warming, *A. floccosus* , as an invasive insect species, has become widespread, causing great ecological interference to the native ecosystem in its new habitat because of its exceptionally high capacity for reproduction in various environmental conditions. By adapting to climate fluctuation, insects tend to move to higher latitudes, altitudes, and inhabit new areas (Bale et al. 2002). Predicting variations in the potential distributions of species has become a primary area of research because of the development of climate models to simulate future climate situations (Annam and Jonathana 2010). Climate change causes insect pest invasions, which cause great damage to commodities and consequential economic losses (Nair and Peterson 2023). Climate change significantly affects the natural enemies, such as predators and parasitoids, of whiteflies, including the woolly whitefly, by changing their development, shortening their lifetime, and causing temporal mismatches in their life cycles associated with the pest (Aregbesola et al. 2018).

The woolly whitefly has a wide host range, with its host plants belonging to 31 plant families; however, citrus is the most preferred host (Evans 2007). In Ethiopia, *the A. floccosus* pest was first reported by Emana (2003) two decades ago from the Central Rift Valley areas of citrus species growing areas. Belay et al. (2011) reported that *A. floccosus* is distributed in central Rift Valley areas of East Shoa, Upper Awash, Melkassa, Dhera, Nazareth, Zeway, Modjo, Meki, Addis Ababa, Debreziet, Bilate (southern Ethiopia), Ataye, Shewrobit, and Kemisie (northern Ethiopia). According to Soliman (2023), *A. floccosus* was documented in Africa after the 1960s, with the first identification in Kenya in 1990. After the year 1980, several southern and eastern African countries, including Tanzania, Rwanda, Burundi, Zambia, Uganda, and Malawi, were reported to be present and then widespread across the African continent. Tello Mercado et al. (2014) reported that it spread in various geographical zones, such as the Nearctic, Neotropical, Palearctic, and Afrotropical. CABI (2021) reported that in Australia, *A. floccosus* is a cosmopolitan species. Furthermore, concerned bodies are organizing biological control agents and integrated pest management systems to limit the climate change‐related risk of *A. floccosus* .

As a limitation of this study, this finding is limited to sweet orange and climatic factors. Further studies are needed to assess other host ranges and other possible factors affecting *A. floccosus* distribution in the country.

## 5 Conclusions

Most of the sweet orange‐growing districts of Ethiopia have hot and humid weather conditions, which strongly favor the growth and development of *A. floccosus* . Among the seven climatic variables, Precipitation of Driest Month (bio14) and Precipitation of Warmest Quarter (bio18) were the two major contributing climatic variables to the potential distribution of *A. floccosus* in Ethiopia. The Maxent model used to predict the very high suitability of *A. floccosus* under current conditions and future RCP2.6, RCP6.0, and RCP8.5 climate change situations indicated that the greater capability of habitat for *A. floccosus* in Ethiopia was chiefly intense in the Central Ethiopia Regional State, large parts of the Oromia Region, the Gambela Region, Dire Dawa city, and the Harari region. Climate change can cause this type of outbreak in cropping systems. Having information of the current and future conditions of the pest serves as a tool to advance strategic policies for *A. floccosus* regulation, inspection, combat, and phytosanitary management. The rapid dispersal ability of the pest associated with climatic variability recorded in Ethiopia, which favors its distribution and development, and researchers recommend that special care should be given to taking effective measures through biological control agents and integrated pest management to prevent further spread of the pest. However, further studies are needed to assess other possible factors affecting *A. floccosus* distribution.

## Funding

This project received funding from the Haramaya University research grant with funding code HURG/2022/01/02/19.

## Ethics Statement

The authors have nothing to report.

## Consent

The authors have nothing to report.

## Conflicts of Interest

The authors declare no conflicts of interest.

## Data Availability

All the required data is provided in the manuscript.
